# dCK negatively regulates the NRF2/ARE axis and ROS production in pancreatic cancer

**DOI:** 10.1111/cpr.12456

**Published:** 2018-04-27

**Authors:** Qiangsheng Hu, Yi Qin, Jinfeng Xiang, Wensheng Liu, Wenyan Xu, Qiqing Sun, Shunrong Ji, Jiang Liu, Zheng Zhang, Quanxing Ni, Jin Xu, Xianjun Yu, Bo Zhang

**Affiliations:** ^1^ Department of Pancreatic Surgery Fudan University Shanghai Cancer Center Shanghai China; ^2^ Department of Oncology Shanghai Medical College Fudan University Shanghai China; ^3^ Shanghai Pancreatic Cancer Institute Shanghai China; ^4^ Pancreatic Cancer Institute Fudan University Shanghai China

**Keywords:** ARE, dCK, gemcitabine, NRF2, pancreatic cancer, ROS

## Abstract

**Objectives:**

Decreased deoxycytidine kinase (dCK) expression is a reported indicator of gemcitabine efficacy in pancreatic cancer, due to the impact of this kinase on gemcitabine metabolism. The transcription factor NF‐E2 p45‐related factor 2 (NRF2, also called Nfe2l2), a master regulator of redox homoeostasis, has been reported to tightly control the expression of numerous ROS‐detoxification genes and participates in drug resistance. However, the contribution of dCK to the NRF2 signalling axis has seldom been discussed and needs investigation.

**Materials and methods:**

By overexpressing dCK in pancreatic cancer cells, we assessed the impact of dCK on NRF2 transcriptional activity. Furthermore, we measured the impact of dCK expression on the intracellular redox balance and reactive oxygen species (ROS) production. By utilizing immunohistochemical staining and tissues from pancreatic cancer patients, we assessed the correlation between dCK and NRF2 expression. Through proliferation and metastasis assays, we examined the impact of dCK expression on cell proliferation and metastasis.

**Results:**

dCK negatively regulates NRF2 transcriptional activity, leading to the decreased expression of ARE‐driven antioxidant genes. In addition, dCK negatively regulates intracellular redox homoeostasis and ROS production. Negative correlations between dCK and NRF2 levels in pancreatic cancer cell lines and patient samples were observed. *In vitro* cell line studies suggested that dCK negatively regulated proliferation and metastasis.

**Conclusion:**

Decreased dCK expression promotes NRF2‐driven antioxidant transcription, which further enhances gemcitabine treatment resistance, forming a feedback loop.

## 1 INTRODUCTION

Despite a low incidence rate, pancreatic cancer remains the fourth leading cause of cancer‐related deaths and is regarded as one of the most malignant and lethal cancer types.^1^, ^2^ Significant progress has been made in the past few decades in solid cancer screening and treatment, which has greatly increased patient chances for a cure. Despite the tremendous progress in pancreatic cancer research, the ratio of mortality to incidence has changed little, and the 5‐year survival rate remains desperately low at approximately 5%‐7%.^3^, ^4^ Surgical resection is considered the only curative treatment for pancreatic cancer. However, most patients have distal organ metastasis at diagnosis, and approximately only 20% of patients have the chance to undergo surgical resection. Thus, chemotherapy treatment or chemotherapy in combination with radiotherapy remains the main option for patients with advanced and metastatic pancreatic cancer.^5^, ^6^

Despite considerable toxicity, 5‐fluorouracil (5‐FU) and its analogs, or combinations thereof, have been widely used for the treatment of advanced pancreatic cancer but are moderately effective at improving a patient's life.^7^ The anti‐cancer agent gemcitabine (2′, 2′‐difluorodeoxycytidine, Gemzar, Eli‐Lilly, Indianapolis, IN) is a cell cycle‐dependent deoxycytidine analog of the antimetabolite class. Since 1997, gemcitabine has been accepted as a reference first‐line therapy drug for patients with a good performance status.^8^ Since then, combinational trials with gemcitabine have been conducted and reported. These combinations included cytotoxic agents (5‐FU, cisplatin, oxaliplatin and capecitabine) and biological agents (erlotinib, Cetuximab and bevacizumab). Although higher clinical benefits and relatively longer survival have been achieved, none of these combination regimens have been proven to be significantly more effective than gemcitabine alone as the first‐line therapy. The overall survival rate remains unchanged.^9^ Gemcitabine has modest clinical benefits and might not improve overall survival to a clinically significant degree due to the inherent chemoresistance of pancreatic cancer cells and the impaired drug delivery system.^10^ Thus, a better understanding of the molecular mechanisms underlying drug resistance in pancreatic cancer is necessary for developing new effective treatments for this lethal disease.

Gemcitabine is a proto‐drug and needs to be taken up and catalysed by a series of enzymes to form the active drug. Gemcitabine is strongly hydrophilic and efficient gemcitabine cell permeation requires specialized integral membrane transport proteins. The major mediators of gemcitabine trafficking are the human equilibrative nucleoside transport (hENT1) and, to a lesser degree, the human concentrative nucleoside transport 3 (hCNT3).^11^, ^12^, ^13^ As a proto‐drug, intracellular gemcitabine must be phosphorylated into its mononucleotide form by deoxycytidine kinase (dCK) for subsequent metabolism. This step is the rate‐limiting step of gemcitabine metabolism. Subsequent nucleotide kinases convert gemcitabine monophosphate to its active metabolites: gemcitabine diphosphate and gemcitabine triphosphate.^14^, ^15^ Gemcitabine exerts its cytotoxicity by blocking de novo DNA synthesis through inhibiting ribonucleotide reductase, which is required for the production of the deoxyribonucleotide precursors needed for DNA synthesis. Ribonucleotide reductase contains a larger subunit, ribonucleotide reductase subunit (RRM)1, and a smaller one, RRM2, that are inactivated by difluorodeoxycytidine‐5‐phosphate.^16^, ^17^ The triphosphorylated form of gemcitabine is incorporated into DNA and leads to chain termination during DNA synthesis. hENT1, dCK and RRM1 are important determinants of gemcitabine activity and gemcitabine‐based chemotherapy efficacy.^18^

Living cells operate optimally within certain pH and temperature ranges; furthermore, the biochemical and physiological processes within a living cell also require an optimal redox balance for the sufficient flux of metabolic processes. The ability of a living cell to adapt rapidly to redox homoeostasis perturbations is essential for survival. Cancerous cells are continuously threatened by ROS and by toxic secondary metabolites generated from ROS‐mediated cell damage, leading to oxidative stress. NRF2 acts as one of the most versatile mechanisms for adapting to cellular oxidative stress and regulates redox homoeostasis to provide proliferative and progressive advantages to cancerous cells.^19^, ^20^ NRF2 plays vital and decisive roles in pancreatic cancer oncogenesis. In a transgenic K‐Ras knock‐in mouse pancreatic ductal adenocarcinoma (PDAC) model with NRF2 simultaneously deleted, pancreatic intraepithelial neoplasia (PanIN), cell proliferation and the tumour burden were reduced.^21^ NRF2 also sustains metabolic reprogramming in cancerous cells. For example, in non‐small cell lung cancer, NRF2 has been reported to regulate serine biosynthesis, providing a growth advantage to cancerous cells.^22^ Highly proliferative cancerous cells require a large quantity of nutrients to maintain high anabolism levels. NRF2 has been reported to be a decisive regulator, redirecting glucose and glutamine anabolism into anabolic pathways, especially under sustained PI3K‐Akt signalling pathway activation, which increases nuclear NRF2 accumulation and NRF2/ARE signalling.^23^ NRF2 overexpression in pancreatic cancer has also been reported to participate in gemcitabine resistance, and inhibiting NRF2 expression and NRF2 transcriptional targets has been reported to improve gemcitabine sensitivity in pancreatic cancer cells.^24^, ^25^ However, the impact of gemcitabine metabolic regulators on the NRF2 signalling pathway has seldom been discussed.

dCK catalyses the rate‐limiting step in gemcitabine metabolism, and a series of studies have demonstrated that decreases in dCK expression, gene mutations, and enzyme activity are important indicators of gemcitabine efficacy. However, the impact of dCK on pancreatic cancer proliferation and the related signalling pathways that might be involved in gemcitabine have seldom been reported. In this study, we demonstrated that decreased dCK expression resulted in hyperactivation of the NRF2/ARE signalling pathway, leading to redox imbalance and increased ROS levels, reinforcing gemcitabine resistance. Moreover, reducing ROS levels by N‐acetyl‐L‐cysteine (NAC) treatment inhibited NRF2/ARE activation and increased dCK expression, promoting gemcitabine sensitivity. In the end, dCK has been demonstrated in vitro to possess tumour suppressive roles in pancreatic cancer cell lines. Collectively, our present study uncovered novel aspects of gemcitabine metabolic regulators in mediating chemotherapy resistance and provided novel intervening strategies.

## 2 MATERIALS AND METHODS

### 2.1 Cell culture

The human pancreatic cancer cell lines PANC‐1 and MIA PaCa‐2 were obtained from the American Type Culture Collection (ATCC, USA). The cells were cultured according to standard protocols provided by ATCC. In brief, PANC‐1 cells were maintained in Dulbecco's modified Eagle's medium (DMEM) supplemented with 10% foetal bovine serum (FBS), 100 U/mL penicillin and 0.1 mg/mL streptomycin. For MIA PaCa‐2 cells, an additional 2.5% horse serum was used in the culture. These cells were maintained in a humidified incubator at 37°C with 5% CO_2_.

### 2.2 Establishment of dCK‐overexpressing cell lines

To overexpress dCK in PANC‐1 and MIA PaCa‐2 cells, a lentivirus‐mediated transfection method was used. The dCK coding sequence was constructed into the pCDH‐CMV‐MCS‐EF1‐Puro lentiviral vector (System Biosciences, Palo Alto, CA, USA). Lentivirus was produced by co‐transfecting dCK‐overexpressing constructs with psPAX2 and pMD2.G vectors at a ratio of 4:3:1 into HEK293T cells. Stable dCK‐overexpressing cell lines were obtained by infecting PANC‐1 and MIA PaCa‐2 cells and subsequent puromycin selection.

### 2.3 Cell viability assay

Cell Counting Kit‐8 (Dojindo, Japan) was used to measure cell viability. Briefly, 200 μL of medium containing cells (3000/well) was added to 96‐well plates. After culturing for the indicated times, CCK‐8 solution was added into each well and incubated at 37°C. After 2 hour, the optical density at 450 nm of each well was measured using a microplate reader.

### 2.4 Cell apoptosis analysis

Flow cytometric techniques were used to measure cell apoptosis. The percentage of apoptotic cells was analysed by fluorescein isothiocyanate‐conjugated Annexin V and propidium iodide (Invitrogen, Carlsbad, CA, USA) staining, followed by flow cytometric analysis.

### 2.5 Quantitative real‐time PCR

Total RNA was extracted using TRIzol reagent (Invitrogen, USA). A TaKaRa PrimeScript RT reagent kit was used for reverse transcription to obtain cDNA (TaKaRa, Japan). The expression status of candidate genes and β‐actin was determined by quantitative real‐time PCR using an ABI 7900HT real‐Time PCR system (Applied Biosystems, Foster City, CA, USA). The primer sequences are listed in Table 1.

**Table 1:** Primers sequences used in the text

dCK forward	5′‐ CAAGACTGGCATGACTGGATGAA ‐3′
dCK reverse	5′‐ GGCACCTCTTGAAGATAATCGAAG ‐3′
GCLM forward	5′‐ ATCTTGCCTCCTGCTGTGTGATGC ‐3′
GCLM reverse	5′‐ CAATGACCGAATACCGCAGTAGCC ‐3′
GCLC forward	5′‐ GTGGTACTGCTCACCAGAGTG ‐3′
GCLC reverse	5′‐ AGCTCCGTGCTGTTCTGGGCCTT ‐3′
ME1 forward	5′‐ CCTCACTACTGCTGAGGTTATAGC ‐3′
ME1 reverse	5′‐ CGGTTCAGGATAAACTGTGGCTG ‐3′
NQO1 forward	5′‐ CGGAGTAAGAAGGCAGTGCTTTC ‐3′
NQO1 reverse	5′‐ TCTGCTGGAGTGTGCCCAATGCT ‐3′
TXNRD forward	5′‐ GCAATCCAGGCAGGAAGATTGCT ‐3′
TXNRD reverse	5′‐ CTCTTGACGGAATCGTCCATTCC ‐3′
HMOX1 forward	5′‐ AGCGGGCCAGCAACAAAGTGCAA ‐3′
HMOX1 reverse	5′‐ CAGCATGCCTGCATTCACATGGC ‐3′
β‐actin forward	5′‐ CCAACCGCGAGAAGATGACCCA ‐3′
β‐actin reverse	5′‐ ATCACGATGCCAGTGGTACG ‐3′

### 2.6 Western blotting

Cells were washed with ice‐cold phosphate‐buffered saline (PBS) and lysed in RIPA buffer (150 mM NaCl, 1% NP‐40, 50 mM Tris/HCl (pH 8.0) and 10% glycerol) containing protease and phosphatase inhibitors purchased from Selleck. Cell debris was removed by centrifugation at 12 000 rpm for 20 minutes at 4°C. The protein concentration of the whole cell lysate was measured using a Thermo Pierce BCA Protein Assay kit. Equal amounts of total protein were separated with SDS‐PAGE and then transferred to PVDF membranes. Antibodies against dCK and NRF2 were purchased from Abcam. The Keap1 antibody was obtained from Proteintech.

### 2.7 Transwell invasion assay

Invasion assays were conducted using a 24‐transwell chamber with a Matrigel‐coated membrane (BD, Franklin Lakes). The lower chamber was filled with 800 μL of media containing 10% FBS. Subsequently, approximately 1 × 10^5^ cells were seeded in 200 μL of medium without serum in the top chamber for the invasion assays. For 24 hour, the cells were incubated at 37°C with 5% CO_2_ and allowed to invade the lower chamber. After removing the non‐migrating or non‐invading cells, the remaining cells were washed, fixed and stained with crystal violet. We counted the number of migrating and invading cells in six fields randomly selected at 100× magnification. Experiments were performed at least in triplicate.

### 2.8 Promoter activity assessment by dual‐luciferase assay

PANC‐1 and MIA PaCa‐2 cells were seeded in 96‐well culture plates and transfected with the indicated vectors using Lipofectamine^™^ 2000 (Invitrogen). The antioxidant NRF2 activity response was assessed using pGMARE‐lu firefly luciferase constructs (Genomeditech, China). The pRL‐TK plasmid (Promega) was used as the internal control. Firefly and Renilla luciferase activities were measured using a dual‐luciferase system (Promega) according to the manufacturer's protocol.

### 2.9 Immunohistochemistry

The clinical tissue samples used in this study were histopathologically and clinically diagnosed at Fudan University Shanghai Cancer Center. Prior patient consent and approval from the Institutional Research Ethics Committee were obtained. Paraffin‐embedded tissue slides were deparaffinized in xylene, rehydrated through graded alcohol solutions, blocked in methanol containing 3% hydrogen peroxide, and then incubated with dCK and NRF2 antibodies. The dCK antibody (Abcam, ab96599) was used at a dilution factor of 1:50. The NRF2 antibody (Abcam, ab62352) was diluted to a ratio of 1:100, and then, the slides were rinsed in PBS solution and incubated with secondary antibodies and peroxidase reagent at room temperature. Finally, the slides were incubated with 3,3′‐diaminobenzidine solution at room temperature for 10 minutes and counterstained with haematoxylin. A scoring scale was used to evaluate the percentage of stained cells (0, <10%; 1, 10%‐25%; 2, 25%‐50%; 3, 50%‐75%; 4, >75%) and the staining intensity (0, negative; 1, low; 2, moderate; 3, strong). The overall staining scores were determined by combining the two scores (frequency × intensity). An immunohistochemical score >6 was defined as high expression, whereas a score ≤6 was considered a low expression level.

### 2.10 ROS measurement and intracellular GSH activity assay

The intracellular ROS level was detected by an oxidant‐sensitive fluorescent probe (DCFH‐DA). Briefly, cells were washed twice with PBS. Then, the cells were stained with 10 μmol/L DCFH‐DA and incubated at 37°C for 20 minutes according to the manufacturer's instruction. Intracellular DCFH‐DA is deacetylated by nonspecific esterases and then is further oxidized by ROS to the fluorescent compound 2,7‐dichlorofluorescein (DCF). DCF fluorescence was detected by a FACScan flow cytometer (Becton Dickinson). Intracellular GSH activity was determined by a GSH/GSSG Ratio Detection Assay kit from Abcam to assess the oxidative status of the pancreatic cancer cells.

### 2.11 Statistical analysis

All data are presented as the means ± SD; experiments were repeated at least three times. Two‐tailed unpaired Student's *t*‐tests and one‐way analysis of variance were used to evaluate the data. SPSS version 16.0 (IBM) was used for the data analysis. Differences were considered significant at *P < .05, **P < .01 and ***P < .001.

## 3 RESULTS

### 3.1 dCK regulates Keap1/NRF2/ARE activation in pancreatic cancer

**Figure 1:**
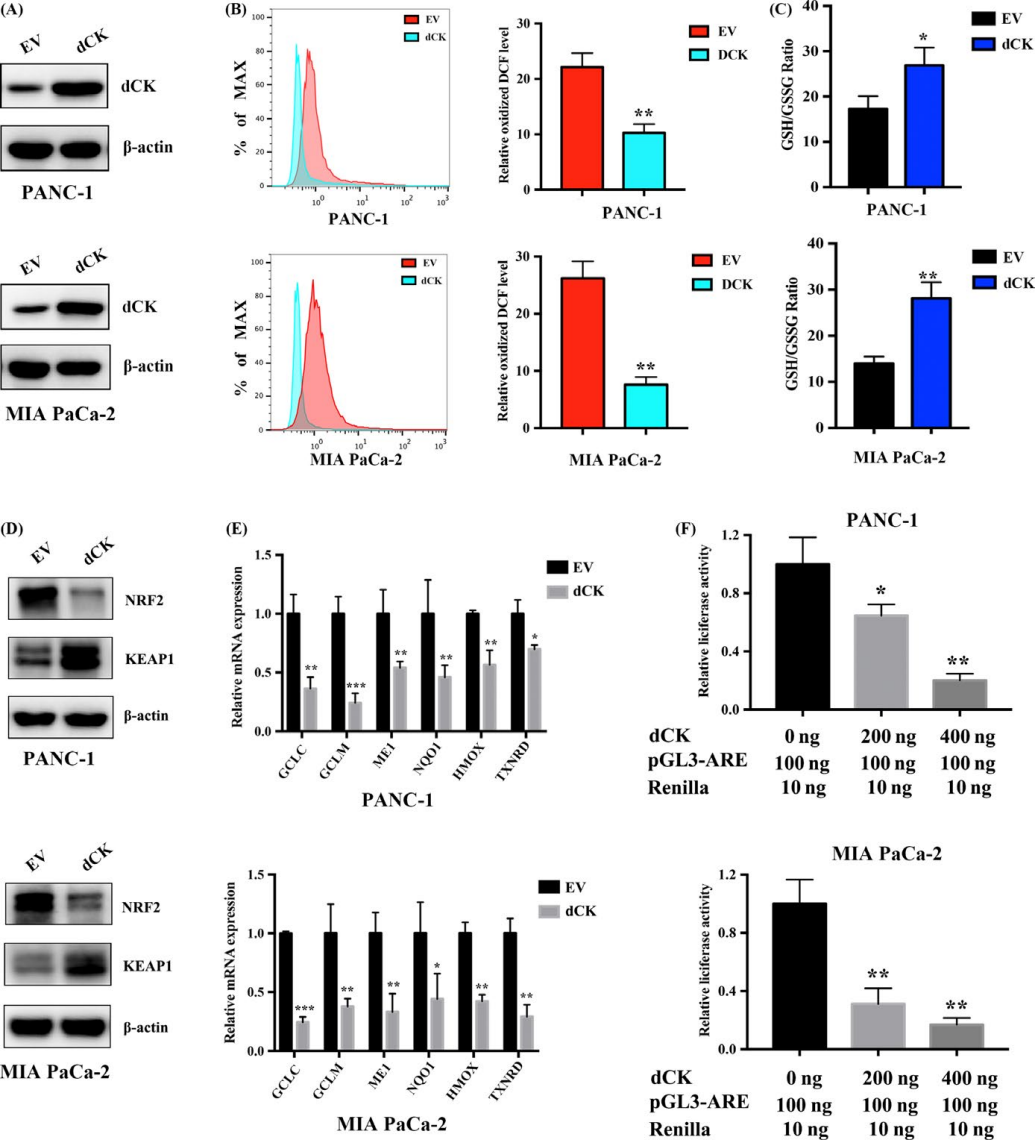
dCK regulates Keap1/NRF2/ARE activation in pancreatic cancer. (A) dCK was introduced into PANC‐1 and MIA PaCa‐2 cells, and the overexpression efficacy was confirmed by immunoblot analysis. (B) dCK overexpression decreased the basal intracellular ROS levels. (C) dCK overexpression increased the GSH/GSSG ratio, leading to a more reduced redox state in PANC‐1 and MIA PaCa‐2 cells. (D) dCK expression decreased the intracellular NRF2 levels and increased the Keap1 protein levels. (E) dCK decreased the expression of ARE‐driven antioxidant genes, including GCLC, GLCM, ME1, NQO1, HMOX and TXNRD. (F) dCK inhibited ARE luciferase activity in a dose‐dependent manner

Decreased dCK expression has been reported to participate in gemcitabine resistance in pancreatic cancer, which is correlated with NRF2/ARE activation. However, the impact of dCK on NRF2/ARE activation has seldom been discussed. First, we overexpressed dCK in PANC‐1 and MIA PaCa‐2 cells, and the overexpression efficacy was validated by western blotting (Figure 1A). Then, we assessed the impact of dCK expression on intracellular ROS production. Through using a reactive oxygen species assay kit, we demonstrated that dCK overexpression decreased intracellular ROS levels in PANC‐1 and MIA PaCa‐2 cells (Figure 1B). Alterations in ROS levels can affect the intracellular redox state, which can be evaluated by the GSH/GSSG ratio. In dCK‐overexpressing PANC‐1 and MIA PaCa‐2 cells, the GSH/GSSG ratio was increased, indicating that dCK might cause a reduced intracellular environment (Figure 1C). NRF2/ARE activation is regarded as a critical regulator of ROS production and redox status in cancer cells. Then, we examined the changes in Keap1 and NRF2 protein levels. As shown, the introduction of dCK increased the Keap1 protein level, while the NRF2 protein levels simultaneously decreased (Figure 1D). NRF2 drives the transcription of a series of genes that participate in ROS detoxification, and the promoter of these genes contains AU‐rich element (ARE) sequences. In dCK‐overexpressing PANC‐1 and MIA PaCa‐2 cells, we observed a decrease in ARE‐driven genes, such as GCLC, GLCM, ME1, NQO1, HMOX and TXNRD (Figure 1E). Finally, we examined the impact of dCK on ARE‐driven luciferase activity. As shown, dCK decreased ARE luciferase activity in PANC‐1 and MIA PaCa‐2 cells (Figure 1F).

### 3.2 dCK suppressed pancreatic cancer cell proliferation

**Figure 2:**
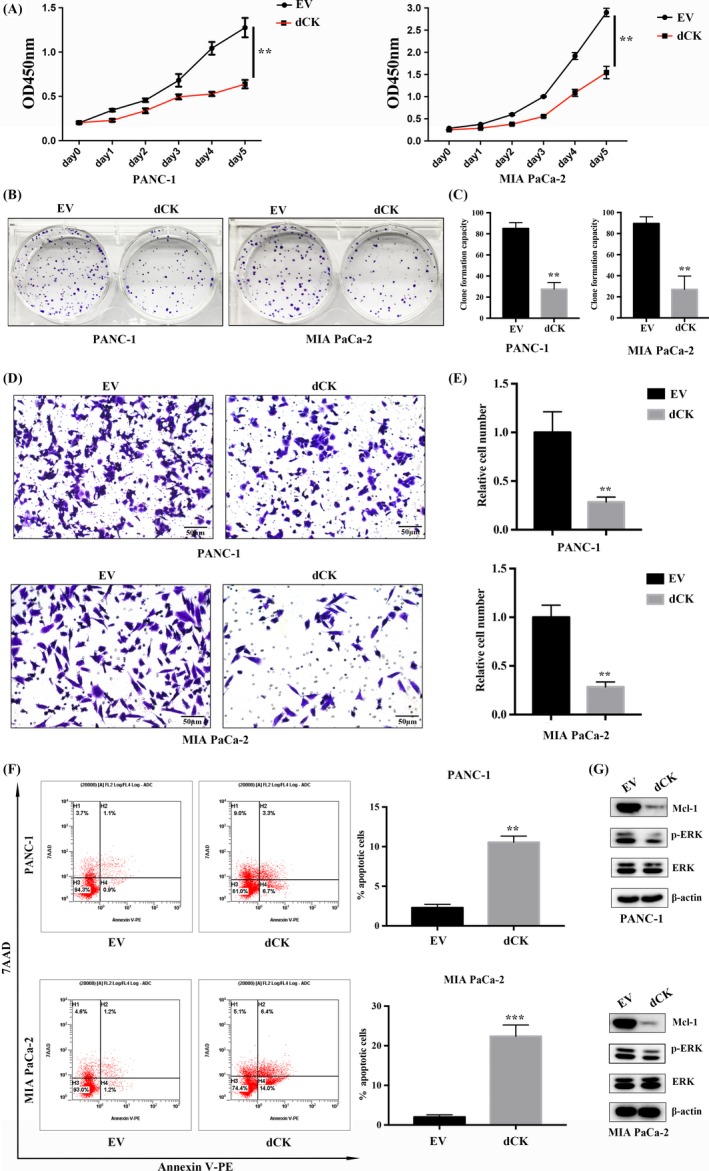
dCK suppressed pancreatic cancer cell proliferation. (A) dCK negatively regulated PANC‐1 and MIA PaCa‐2 cell viability. (B) and (C) dCK overexpression inhibited the colony formation capacity of PANC‐1 and MIA PaCa‐2 cells. (D) and (E) dCK inhibited PANC‐1 and MIA PaCa‐2 cell invasiveness. (F) DCK overexpression increased apoptosis in PANC‐1 and MIA PaCa‐2 cells. (G) dCK inhibited ERK1/2 activation and decreased the expression of the anti‐apoptotic factor Mcl1 in pancreatic cancer cells

On the basis of our observations of the negative correlation between dCK and NRF2 expression and the decisive roles of NRF2 in pancreatic cancer oncogenesis and progression, we proposed that dCK might inhibit pancreatic cancer cell proliferation. First, we performed a CCK‐8 proliferation assay, and the results suggested that dCK overexpression inhibited PANC‐1 and MIA PaCa‐2 cell proliferation (Figure 2A). Next, we performed colony formation assays, and the results indicated that dCK overexpression suppressed the colony formation capacity of PANC‐1 and MIA PaCa‐2 cells (Figure 2B,C). Then, we assessed the impact of dCK expression on PANC‐1 and MIA PaCa‐2 cell invasiveness and observed that dCK overexpression inhibited the invasive capacity of PANC‐1 and MIA PaCa‐2 cells (Figure 2D,E). Next, we analysed apoptosis by flow cytometry and found that dCK overexpression promoted apoptosis in PANC‐1 and MIA PaCa‐2 cells, indicating a negative role for dCK in pancreatic cancer proliferation (Figure 2F). Finally, we analysed the potential pathways that participate in drug resistance and anti‐apoptosis. Our results demonstrated that dCK overexpression inhibited ERK1/2 activation. In addition, the protein levels of Mcl1, a well‐characterized anti‐apoptotic factor that participates in chemotherapy and radiotherapy resistance, were also decreased (Figure 2G).

### 3.3 Decreased dCK expression and activation of the NRF2/ARE axis are observed in gemcitabine‐resistant cells

**Figure 3:**
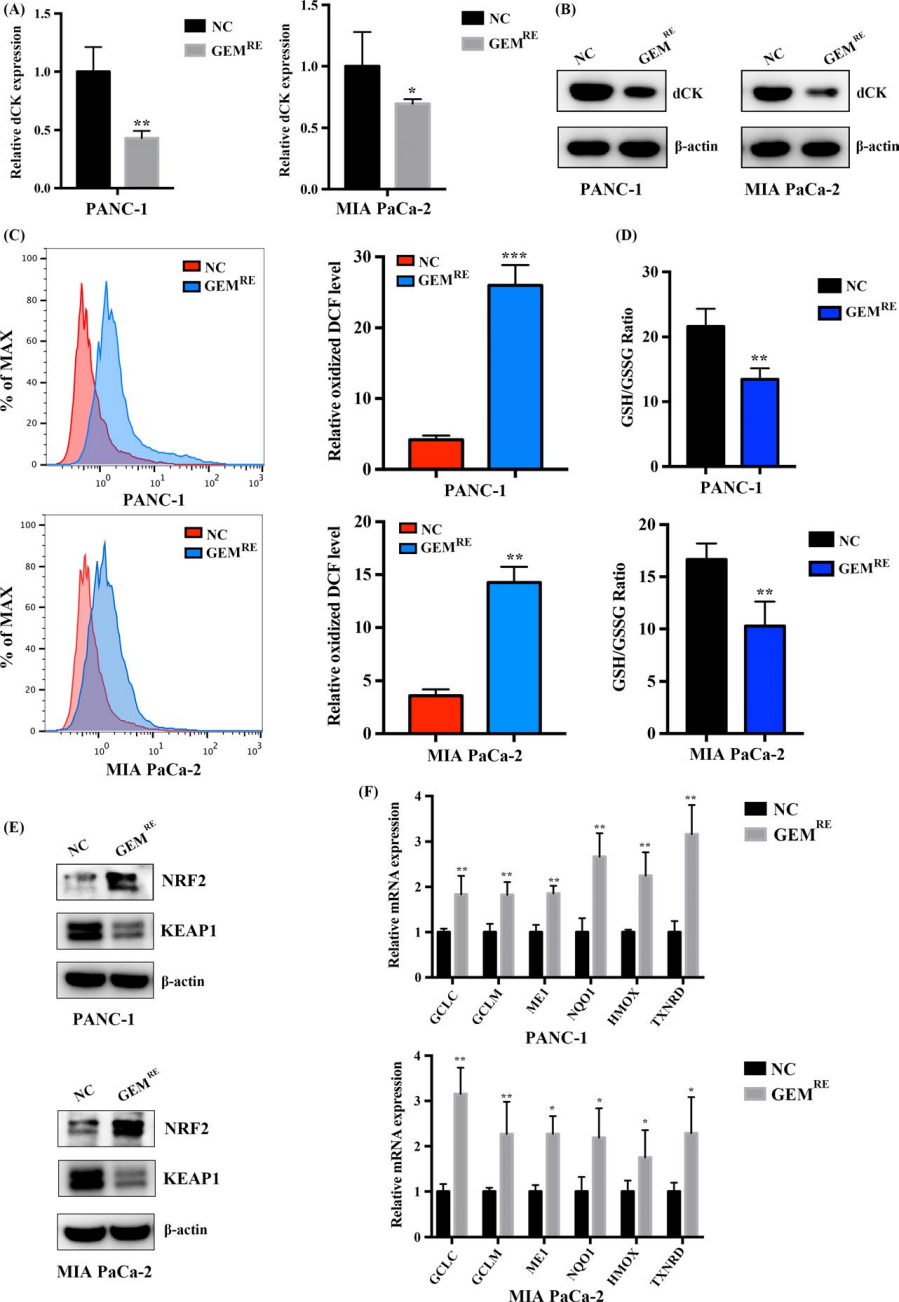
Decreased dCK expression and NRF2/ARE axis activation were observed in gemcitabine‐resistant cells. (A) and (B) The transcript and protein levels of dCK were decreased in gemcitabine‐resistant PANC‐1 and MIA PaCa‐2 cells. (C) The intracellular ROS levels were significantly higher in gemcitabine‐resistant PANC‐1 and MIA PaCa‐2 cells. (D) The GSH/GSSG ratio was significantly lower in gemcitabine‐resistant PANC‐1 and MIA PaCa‐2 cells, indicating an oxidized intracellular microenvironment. (E) In gemcitabine‐resistant PANC‐1 and MIA PaCa‐2 cells, NRF2 protein levels were higher, and the protein levels of Keap1 were decreased. (F) NRF2‐driven, ROS‐detoxification genes were increased in gemcitabine‐resistant PANC‐1 and MIA PaCa‐2 cells

As observed above, dCK regulates the Keap1/NRF2/ARE axis in pancreatic cancer cells. We propose that dCK expression might be negatively correlated with the NRF2/ARE axis in gemcitabine‐resistant cells. In gemcitabine‐resistant PANC‐1 and MIA PaCa‐2 cells, we observed a decrease in dCK mRNA and protein levels (Figure 3A,B). Then, we measured the intracellular ROS levels and observed an increased ROS level in the gemcitabine‐resistant PANC‐1 and MIA PaCa‐2 cells (Figure 3C). Next, we measured the intracellular GSH/GSSG ratio to assess the intracellular redox status, and the results indicated that the GSH/GSSG ratio was significant lower in the gemcitabine‐resistant cells than in the parent cells, indicating that gemcitabine resistance might correlate with redox balance (Figure 3D). Furthermore, we assessed Keap1 and NRF2 expression in gemcitabine‐resistant PANC‐1 and MIA PaCa‐2 cells and observed a decrease in Keap1 protein levels and an increase in NRF2 levels (Figure 3E). Finally, we assessed the expression of NRF2‐targeted, ARE‐driven ROS‐detoxification genes and observed a significant increase in ARE‐driven gene levels in the gemcitabine‐resistant cells (Figure 3F).

### 3.4 NAC treatment increases dCK expression and improves cell sensitivity to gemcitabine

**Figure 4:**
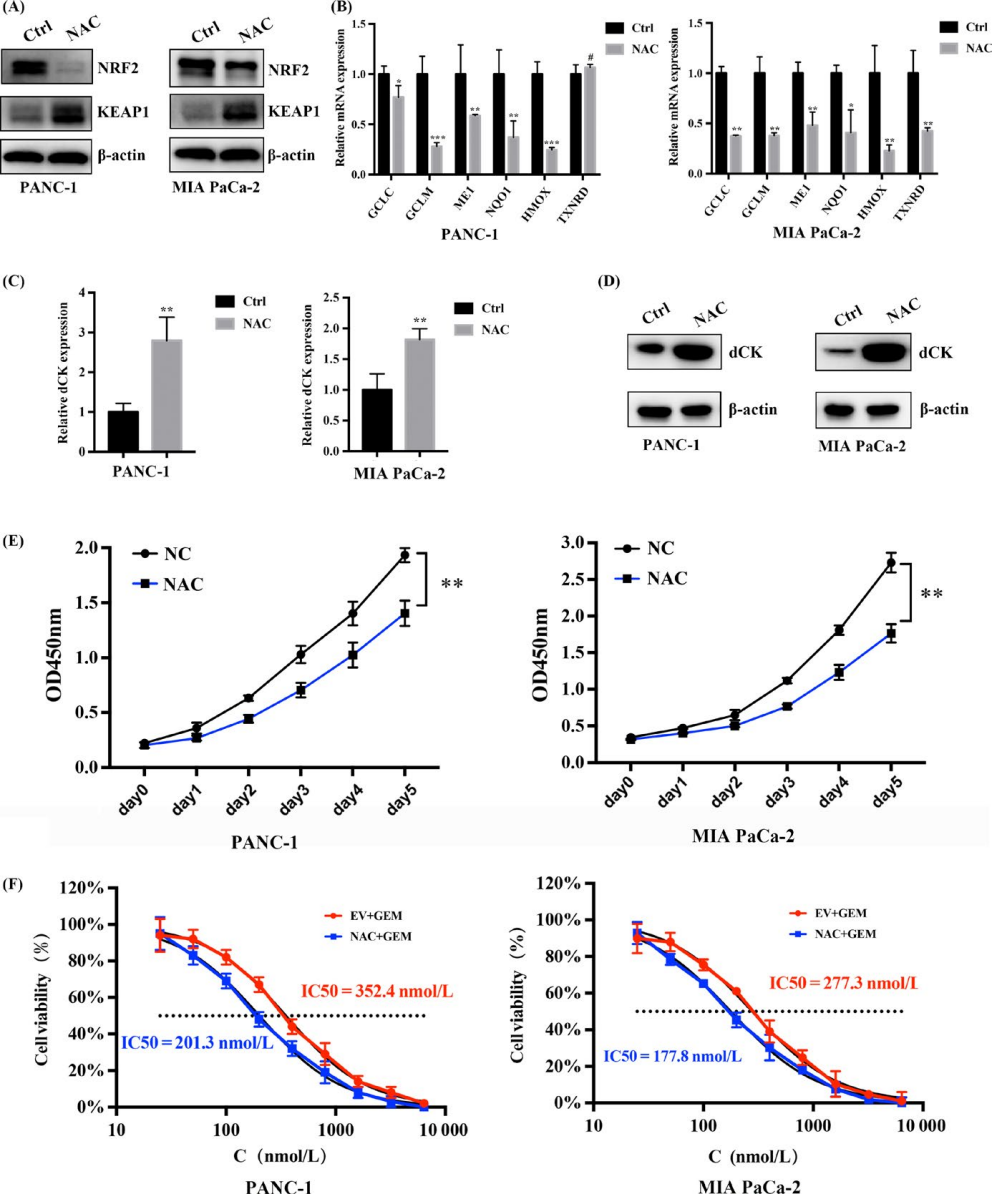
NAC treatment increases dCK expression and promotes cell sensitivity to gemcitabine. (A) NAC treatment increased the Keap1 protein levels, and meanwhile, the protein levels of NRF2 decreased. (B) NAC treatment decreased the expression of NRF2‐targeted, ARE‐driven genes. (C) and (D) NAC treatment (10 mmol/L NAC) increased dCK mRNA and protein expression levels. (E) Treatment of PANC‐1 and MIA PaCa‐2 cells with NAC inhibited cell proliferation. (F) NAC treatment in PANC‐1 and MIA PaCa‐2 cells decreased the IC50 of gemcitabine, indicating a positive role for NAC in gemcitabine efficacy

In cells, NAC is frequently used as a sulfhydryl source and acetylated precursor for reduced GSH. NAC also interacts directly with ROS and scavenges oxygen free radicals. Thus, we treated PANC‐1 and MIA PaCa‐2 cells with NAC to inhibit intracellular ROS activity and to examine the subsequent impact on dCK expression. We first examined the impact of NAC treatment in PANC‐1 and MIA PaCa‐2 cells on Keap1 and NRF2 expression and observed a decrease in NRF2 levels and an increase in Keap1 levels (Figure 4A). Next, we assessed the impact of NAC on the expression of ARE‐driven ROS‐detoxification genes and observed a decrease in these gene levels, suggesting a depressive role for NAC on the Keap1/NRF2/ARE axis (Figure 4B). Then, we assessed the impact of NAC treatment on dCK expression to prove whether the intracellular ROS status regulates dCK expression. As shown, the 10 mmol/L NAC treatment in PANC‐1 and MIA PaCa‐2 cells increased dCK mRNA and protein expression levels, indicating that intracellular ROS production might regulate dCK expression (Figure 4C,D). Next, we performed cell proliferation assay to examine the impact of NAC on cell proliferation of PANC‐1 and MIA PaCa‐2 cells. As observed, treatment of PANC‐1 and MIA PaCa‐2 cells with 10 mmol/L of NAC could inhibit proliferation(Figure 4E). Due to the important roles of dCK on gemcitabine resistance, we measured the impact of NAC treatment on gemcitabine sensitivity, and our results indicated that NAC treatment decreased the IC50 values and increased cell sensitivity to gemcitabine in PANC‐1 and MIA PaCa‐2 cells (Figure 4F).

### 3.5 dCK is negatively correlated with NRF2 expression in pancreatic cancer patients

**Figure 5:**
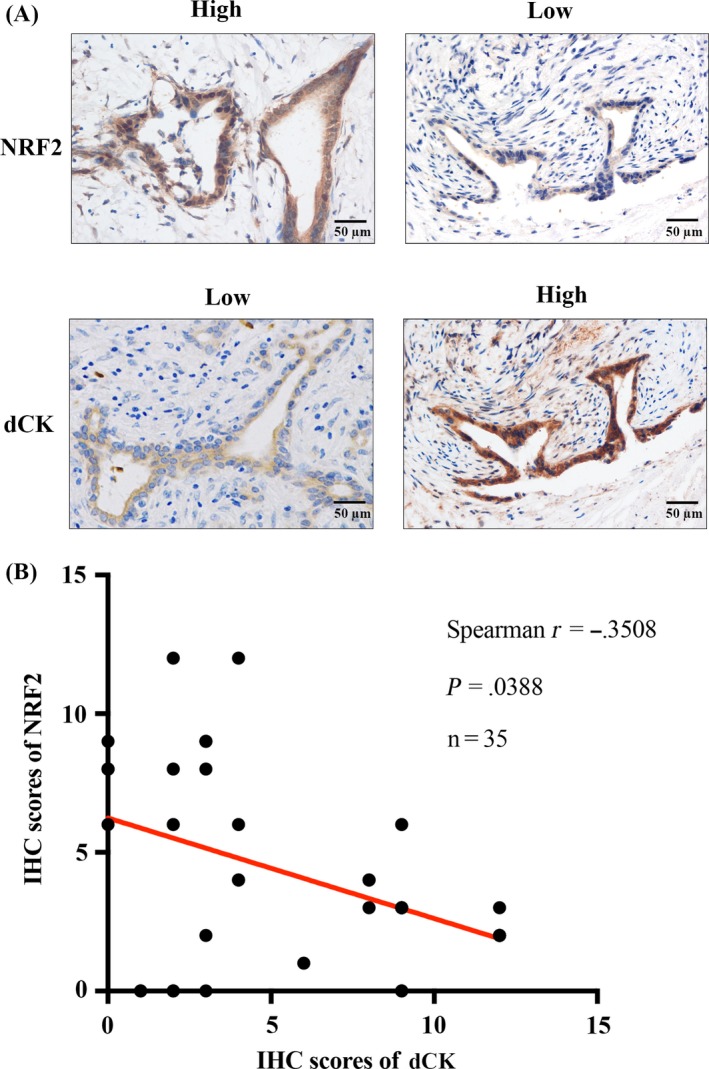
dCK expression is negatively correlated with NRF2 levels in pancreatic cancer patients. (A) Patients with higher dCK levels exhibited lower levels of NRF2, while NRF2 expression was higher in patients with lower dCK levels. (B) dCK was negatively and significantly correlated with NRF2 expression in pancreatic cancer patients

As discussed above, we observed a negative correlation between dCK and NRF2 expression in vitro in pancreatic cancer cell lines. Next, we examined dCK and NRF2 expression in pancreatic cancer patients. As shown, patients with lower dCK levels exhibited higher levels of NRF2, indicating a negative correlation between these two proteins (Figure 5A). Next, we increased the number of patient cases, and performed IHC staining to measure the correlation between dCK and NRF2 expression. In addition, the statistical analysis indicated that dCK expression is negatively and significantly correlated with NRF2 expression in pancreatic cancer patients (Figure 5B).

**Figure 6:**
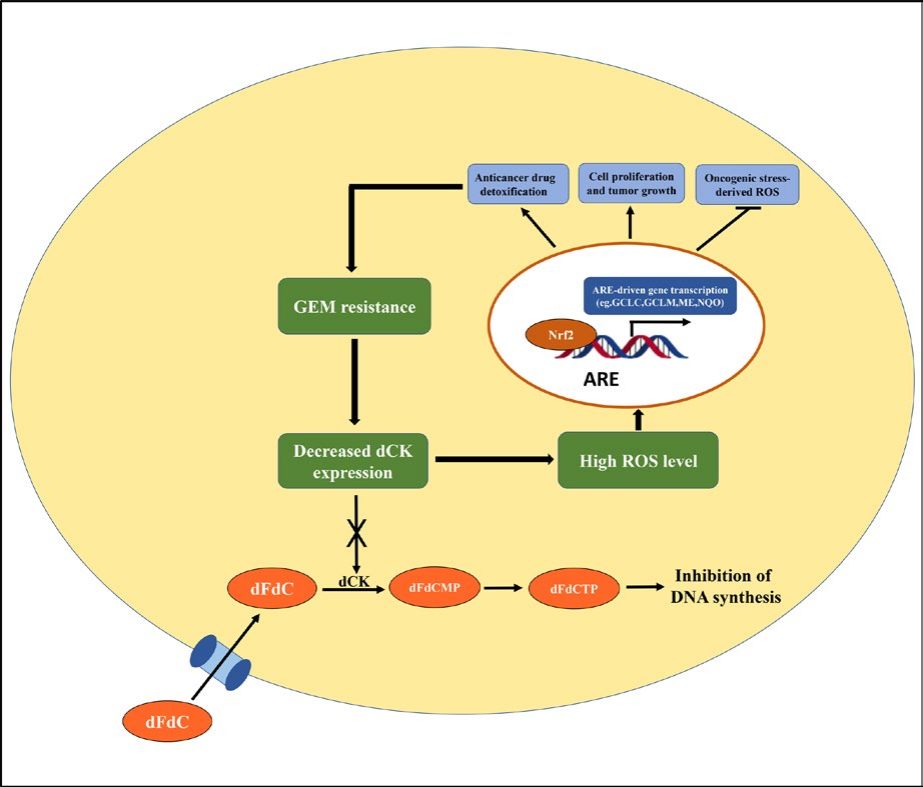
Schematic representation of the working model. Decreased dCK expression contributes to gemcitabine resistance. Furthermore, low dCK levels also activated the NRF2/ARE axis, leading to increased ROS levels, which also lead to gemcitabine resistance. Thus, a dCK‐NRF2/ARE feedback loop exists, which collectively renders pancreatic cancer cells gemcitabine resistant

In conclusion, our present study identifies the negative impact of the gemcitabine metabolic regulator dCK on the Keap1/NRF2/ARE axis and reveals that decreased dCK expression regulates ROS production and the intracellular redox status, which might contribute to gemcitabine resistance and regulate pancreatic cancer cell proliferation (Figure 6).

## 4 DISCUSSION

Although significant progress has been made in the diagnosis and treatment of pancreatic cancer, this disease remains as one of the most lethal cancer types and has the worst survival rate of all cancers. The current treatment standard for metastatic pancreatic cancer is gemcitabine‐based chemotherapy; however, the efficacy of this treatment is poor, and overall survival has not improved for decades. Both intrinsic and acquired drug resistance are major reasons for the unsatisfying results in patients. Thus, exploring the underlying molecular mechanism that governs drug resistance might help improve overall survival in pancreatic cancer patients.

dCK is a key enzyme that catalyses the process of deoxyribonucleoside salvage, which plays important roles in maintaining normal DNA metabolism. dCK can also activate many antiviral and anti‐cancer nucleoside analogs, such as fludarabine, gemcitabine, cladribine and zalcitabine. In pancreatic cancer, dCK catalyses gemcitabine activation, and decreased dCK expression is considered an important factor governing gemcitabine resistance. Decreased dCK levels contribute to gemcitabine resistance by reducing the level of the active gemcitabine form.^26^ dCK also participates in DNA damage and repair, a process that contributes for radiotherapy resistance.^27^ However, the impact of dCK on signal transduction in cancer cells has seldom been documented. Uncovering the signalling pathways affected by dCK might provide novel strategies for improving chemotherapy and radiotherapy in pancreatic cancer. Many signalling pathways have been reported to regulate gemcitabine resistance, and among them, the NRF2/ARE signalling axis has received the most attention. Gemcitabine stimulates ROS generation in pancreatic cancer cells, resulting in constitutive NRF2 activation, leading to intrinsic gemcitabine resistance.^28^ Thus, gemcitabine metabolic enzymes might also possess certain roles in ROS generation and the NRF2/ARE axis. In our present study, we demonstrated that dCK suppresses ROS generation and inhibits NRF2 activation, a phenomenon that had seldom been reported before. On the basis of this observation, we deduced that in addition to the role of dCK in gemcitabine metabolism, this kinase may also contribute to drug resistance by regulating ROS production and the NRF2/ARE axis, which synergistically regulate intrinsic and acquired gemcitabine resistance in pancreatic cancer cells. Previous studies have demonstrated that NRF2 activation is the net result of oncogenic Kras mutation and MAPK pathway activation.^29^ Consistent with this finding, we also observed a decrease in ERK1/2 activation in dCK‐overexpressing PANC‐1 and MIA PaCa‐2 cells. Gemcitabine‐induced MAPK signalling is a key cause of chemotherapy resistance, and inhibiting MAPK signalling pathways with Erlotinib prolongs pancreatic cancer patient survival.^30^ Thus, targeting dCK to inhibit the resultant ERK1/2 activation and NRF2/ARE axis might provide novel treatment targets for metastatic pancreatic cancer.

Based on the above reports and discussions, the intracellular dCK level is a promising target in pancreatic cancer. However, the regulatory mechanisms of dCK have seldom been discussed in pancreatic cancer. In idiopathic pulmonary fibrosis (IPF), dCK has been reported to be a downstream target of hypoxia and contributed to alveolar epithelial cell proliferation.^31^ In chronic obstructive pulmonary disease (COPD), dCK has been also reported to be induced by hypoxia, and increased dCK levels contribute to apoptosis in chronic lung disease.^32^ Hypoxia inducible factor 1α (HIF1α) is a master regulator of the hypoxic response and acts as a transcription factor that governs the expression of many hypoxia‐induced genes.^33^ The transcription factors SP1 and USF have been reported to bind to the dCK promoter and regulate dCK transcription in cancer cells.^34^, ^35^ However, direct dCK transcription by HIF1α has seldom been discussed. Previous studies have demonstrated that HIF1α is tightly regulated by intracellular ROS levels, and increased ROS generation stabilizes HIF1α protein levels.^36^ In our study, we also observed that scavenging ROS production by NAC increased dCK expression. Moreover, using PROMO 3.0 to identify potential transcription factors, Blackburn MR reported that potential HIF1α binding sites exist in the dCK promoter region. Furthermore, potential p53 and NF‐κB binding sites also exist in the dCK promoter.^32^ The transcriptional activities of p53 and NF‐κB are also under ROS regulation, indicating that the intracellular ROS levels and redox balance might govern dCK transcript expression.^37^, ^38^

The dCK levels were also regulated at post‐transcriptional levels. One recent study demonstrated that ROS detoxification and microRNA (miR)‐155 suppressed post‐transcriptional dCK levels, leading to chemoresistance in pancreatic cancer cells.^39^ Post‐translational modifications also affect dCK enzymatic activity, regulate drug metabolism and contribute to drug resistance in cancer. For example, ataxia telangiectasia mutated (ATM) phosphorylates and activates dCK at serine 74 in response to ionizing radiation (IR). dCK activation shifts dCK substrate specificity towards deoxycytidine, increases the intracellular dCTP pools and DNA repair activity, and contributes to chemotherapy and radiotherapy resistance.^27^, ^40^ dCK phosphorylation at serine 74 is reversed by protein phosphatase 2A, which negatively regulates dCK activity.^41^ Moreover, using a mass spectrometry technique, dCK was found to exist in a complex that contains cyclin‐dependent kinase 1 (Cdk1). After IR, Cdk1 interacts with dCK, and the activity of Ckd1 is inhibited by dCK both in vitro and in vivo, making dCK an important G2/M checkpoint regulator in response to DNA damage.^42^ Increased basal ROS levels can induce apoptosis, and one possible mechanism for this effect is that increased basal ROS levels activate ATM.^43^, ^44^ Thus, increased basal ROS levels might impact the post‐translational modification of dCK, participating in cell apoptosis. However, the impact of gemcitabine on dCK post‐translational modifications has seldom been studied, and further studies are needed to shed light on dCK post‐translational modifications, especially under the context of gemcitabine resistance.

## 5 CONCLUSIONS

In conclusion, our present study uncovered novel roles for the gemcitabine metabolic enzyme dCK in ROS detoxification and NRF2/ARE transcription. In addition, our studies also demonstrated that scavenging intracellular ROS increased dCK mRNA and protein levels. Together with previous dCK reports, we have increased the understanding of the role of this enzyme in pancreatic cancer and have shed light on novel strategies for improving chemotherapy resistance in pancreatic cancer.

## ACKNOWLEDGEMENTS

This work was supported by the National Natural Science Foundation (81502031, 81372651 and 81602085); National Science Fund for Distinguished Young Scholars (81625016); and Shanghai Sailing Program (16YF1401800).

## CONFLICTS OF INTEREST

No potential conflicts of interest were disclosed.
